# Mapping Maternal Health in the New Media Environment: A Scientometric Analysis

**DOI:** 10.3390/ijerph182413095

**Published:** 2021-12-11

**Authors:** Yinghua Xie, Dong Lang, Shuna Lin, Fangfei Chen, Xiaodong Sang, Peng Gu, Ruijun Wu, Zhifei Li, Xuan Zhu, Lu Ji

**Affiliations:** 1 School of Medicine and Health Management, Tongji Medical College, Huazhong University of Science and Technology, Wuhan 430030, China; m202078149@hust.edu.cn (Y.X.); langdong@hust.edu.cn (D.L.); linshuna@hust.edu.cn (S.L.); chenfangfei@hust.edu.cn (F.C.); 2 Research Center for Rural Health Service, Key Research Institute of Humanities and Social Sciences of Hubei Provincial Department of Education, Wuhan 430030, China; 3 China Biotechnology Development Center, Beijing 100039, China; sangxd@cncbd.org.cn (X.S.); wurj@cncbd.org.cn (R.W.); lizf@cncbd.org.cn (Z.L.); 4 China Science and Technology Exchange Center, Beijing 100045, China; gupeng8718@163.com; 5 School of Computer, Central China Normal University, Wuhan 430079, China

**Keywords:** maternal health, new media, bibliometric analysis, cited reference analysis, structural variation analysis, hotspots

## Abstract

Background: The new media provides a convenient platform to access, use and exchange health information. And as a special group of health care, maternal health care is still of international concern due to their high mortality rate. Scientific research is a good way to provide advice on how to improve maternal health through stringent reasoning and accurate data. However, the dramatic increase of publications, the diversity of themes, and the dispersion of researchers may reduce the quality of information and increase the difficulty of selection. Thus, this study aims to analyze the research progress on maternal health under the global new media environment, exploring the current research hotspots and frontiers. Methods: A scientometric analysis was carried out by CiteSpace5.7.R1. In total, 2270 articles have been further analyzed to explore top countries and institutions, potential articles, research frontiers, and hotspots. Results: The publications ascended markedly, from 29 in 2008 to 472 publications by 2020. But there is still a lot of room to grow, and the growth rate does not conform to the Price’s Law. Research centers concentrated in Latin America, such as the University of Toronto and the University of California. The work of Larsson M, Lagan BM and Tiedje L had high potential influence. Most of the research subjects were maternal and newborn babies, and the research frontiers were distributed in health education and psychological problems. Maternal mental health, nutrition, weight, production technology, and equipment were seemingly hotspots. Conclusion: The new media has almost brought a new era for maternal health, mainly characterized by psychological qualities, healthy and reasonable physical conditions and advanced technology.

## 1. Introduction

The WHO defines maternal fitness as women’s health care during pregnancy, childbirth, and postpartum. However, more than half a million women die each year from pregnancy globally, mostly in developing countries [1]. Therefore, improving maternal health was proposed as one of Millennium Development Goals to lessen maternal and infant mortality [2]. Governments worldwide responded positively, not only by issuing a series of policies but also by launching some national actions. Laos and Nepa l [3,4] have implemented free maternal health policies, and China and Cambodia have enrolled maternal healthcare in essential public health services [5,6]. Maternity hospitals in Uzbekistan and Kyrgyzstan reserved skilled obstetric talents to provide maternal services [7]. Brazil promulgated respectively National Programme in 2000 and 2004, which had significantly reduced maternal mortality, child mortality, and stunting in children under five years of age [8].

Some progress has been made in the academic community [5,9,10]. For instance, researchers have been making thorough inquiries into the factors affecting the utilization of health services for maternal [11] and the association between maternal and infant health [12]. New media is a relatively broad social media based on information technology and carried by consumer electronics. The new media environment, a collection of many media, has become a part of the social environment and attracted lots of researchers’ interest, due to it has greatly enriched the access to health information compared to traditional ways such as follow-up services [13,14]. However, whether pregnant women could effectively use the information, it still depend on certain health literacy [15], which is the synthesis of health belief and health behavior based on health knowledge [16].

In a nutshell, the rapid development of new media has provided convenience to find health information [17]. According to the data of Internet World Statistics (IWS), in the first quarter of 2021, there were 7.8 billion Internet users worldwide, accounting for 65.6% of the world’s population [18]. As an important intermediary to communicate health information, the new media not only provides mass data for scientific research [18], but also expands a new dimension to improve the effectiveness of medical care [19,20]. However, previous studies revealed that the abundance and complexity of health information in the new media environment may reduce maternal coherence [21] and result in certain risks compared to traditional interventions for maternal health [22].At present, a few publications focus on the new media or pregnant women’s health using the scientometric method, and none publication conduct structural variation analysis to explore the impact of the new media environment on maternal health. CiteSpace, as a well-known and free scientometric software characterized by vitality and intuition, is widely used in various specific research fields [23]. Therefore, this study is going to employ CiteSpace5.7.R1 (Chaomei Chen, Philadelphia, PA, USA) to analyze maternal health in the new media environment, aiming at understanding the status and summarizing the current hot topics, and laying a solid foundation for improving maternal health research work.

## 2. Methods

### 2.1. Recruitment

We defined the topic of this study as maternal health in the environment of new media. We extracted three keywords in exploring specific definitions: pregnant women, new media, and health. By reading the previous literature, we preliminarily determined several definitions regarding pregnant women, which helps us understand pregnant women’s complicated nature. Besides, this study enriches and improves the expressions of synonyms by using MeSh Browser. Paralleling with operating the concept of pregnant women, we also use a substantial array of new media as part of the search strategy. Overall, The search strategy combines free-text terms such as maternity, health, and new media: “TS = (‘pregnant woman’ OR ‘expectant mother’ OR ‘lying-in woman’ OR puerpera OR ‘pregnant and lying-in woman’ OR parturient OR ‘delivery woman’ OR puerperal OR primipara OR ‘woman expecting confinement’ OR ‘gestational woman’ OR gravida OR maternal OR ‘pregnant and birth-giving woman’) AND TS = (Online OR ‘social media’ OR web OR virtual OR cyber OR Orkut OR Twitter OR Facebook OR Reddit OR Instagram OR Snapchat OR youtube OR Whatsapp OR WeChat OR QQ OR Tumblr OR Linkedin OR Pinterest) AND TS = (health*).”

**Figure 1:**
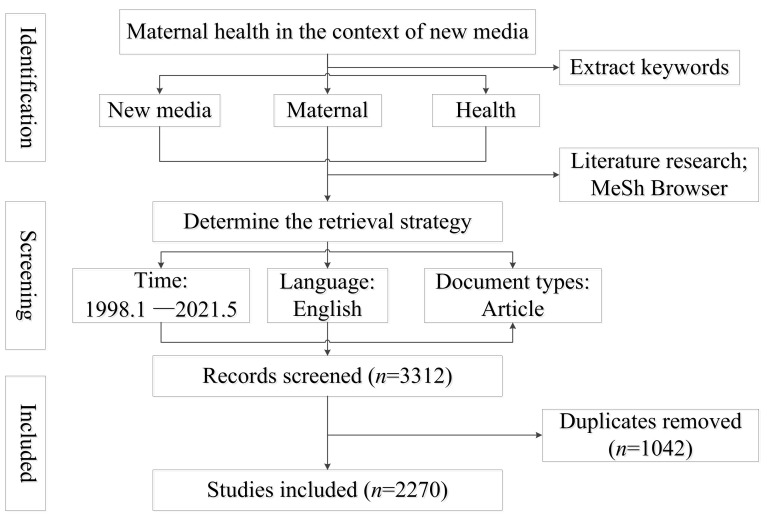
Flow diagram of publications search.

Due to the remarkable interdisciplinary characteristics of publications that existed in the academic community, the most widely used [24] the core database of Web of Science was chosen as the data source. Generally, articles were cited more frequently than other studies and were second only to meta-analysis, but also gave new research conclusions [25]. For clarity on the research status about maternal health for nearly 20 years in the new media environment, therefore we systematically searched the articles in the core database of Web of Science between January 1998 and May 2021. Finally, 3312 publications were included in CiteSpace to analyze the research trends, summarize the current research hotspots and frontiers of maternal fitness in the new media environment. The data retrieval and selection are displayed in Figure 1.

### 2.2. Mapping the Knowledge Map

As a combination of science and art, knowledge maps help us detect the structure of knowledge about a specific area. CiteSpace is facilitated to reveal the structure and dynamics of a knowledge domain graphically. In this study, CiteSpace V. 5.7.R1(64-bit) was used for scientometrics analysis. Firstly, 3312 publications retrieved were imported into CiteSpace. After removing duplicates, in a total of 2270 publications were eventually included in further analysis. The specific parameters were set as follows: The time slice was set as three years, then the study period was divided into nine partitions. Term source consists of titles, abstracts, and keywords. The connection strength was set as cosine. The node threshold value was “Top50”, that is, each slice consists of 50 documents with the highest citation frequency. Network pruning is allowed, selecting "Pruning path." The visual view mainly selects co-occurrence view, static clustering view, and timeline view. The analysis of the top countries, institutions, cited references, and keywords regarding maternal health research in the new media environment were conducted to explore the current research frontiers and hotspots.

In the co-occurrence view, the node’s size indicates the frequency of occurrence of fields (such as country, institution, keyword). The color of the node (citation ring) represents the field’s history. The thickness of the color and the corresponding time is proportional. The color changes from cold to warm. The line between nodes represents the relationship between nodes. Notably, the thicker the line, the closer the relationship is. Meanwhile, the centrality could predicts transformative changes, it could show more valuable information than the indicator of frequency. The calculation of centrality is as follows [26]: $BC_{i}=\sum_{\begin{array}{c} s\neq \;i\;\neq \;t \end{array}}\frac{n_{st}^{i}}{g_{st}}$, $g_{st}$ is the amount of shortest paths from S to T, and $n_{st}^{i}$ is the quantity of shortest ways passing through node I in $g_{st}$. In general, emerging scientific discoveries will have high centrality [27]. Nodes with purple circles have higher centrality (≥0.1), and the thicker purple aperture indicates greater importance. In addition, burst detection could visually reflect the rise and fall of a particular subject in CiteSpace [28]. In the cluster view, the smaller the cluster number, the larger the cluster. The clustering color is consistent with the clustering label color. The timeline view could reflect the clustering results and visually compare the period of clustering duration. Every publication or keyword with a high burst represents a breakthrough in the existing network structure.

## 3. Results

### 3.1. Publications Analysis

**Figure 2:**
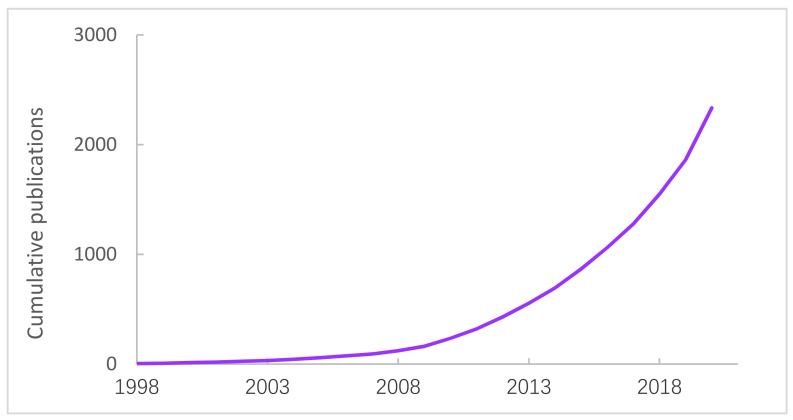
Cumulative Publications Curve from 1998 to 2021.

**Figure 3:**
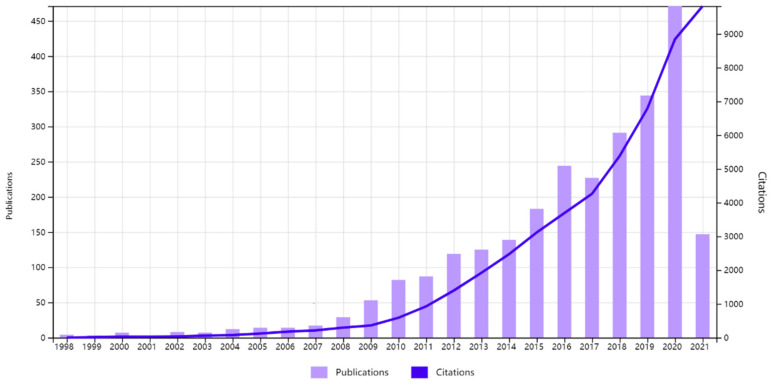
Times Cited and Publications from 1998 to 2021.

Research on maternal fitness in the new media environment started in 1998, with the publications and citation activities increased over the year (R = 0.9883, *p* < 0.05). After further verification, it was found that this growth does not conform to Price’s law, because the exponential fitting line was similar to the moving average (Figure 2). Nevertheless, the growth was different, which could be divided into two stages. From 1998 to 2007, the number of publications and citation activity was relatively small, with relatively slow growth. There was steady and rapid growth in publications and citation activities between 2008 and 2020. The number of 472 publications in 2020 was more than 16 times in 2008, showing a remarkable ascend (Figure 3).

### 3.2. Top Country and Institution

**Figure 4:**
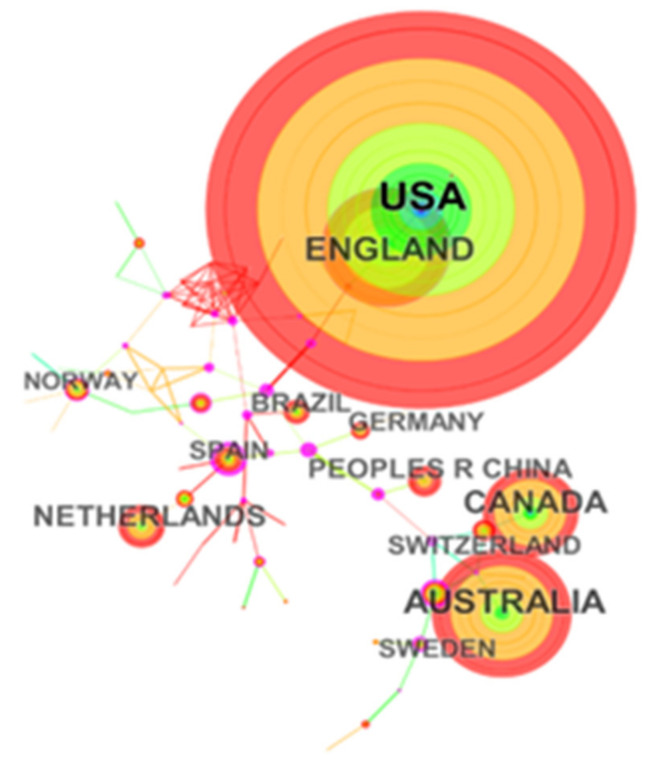
Collaborative network-based countries.

The 2270 articles on maternal fitness in the new media environment were published by study groups in 72 countries (Figure 4). The top 10 countries (five located in Europe, three in American, one in Asia and Australia) published 2184 articles, accounting for 96.21% of the total publications. The United States had the most significant publications, with 905 publications, accounting for 39.87%. Australia, The United Kingdom, Canada, and the Netherlands followed. China had published 84 papers in academics, accounting for 3.70%, ranking sixth. In this research field, the United States kept close cooperation with the United Kingdom, so were Australia and Canada. At present, this field has three significant clusters. 

**Figure 5:**
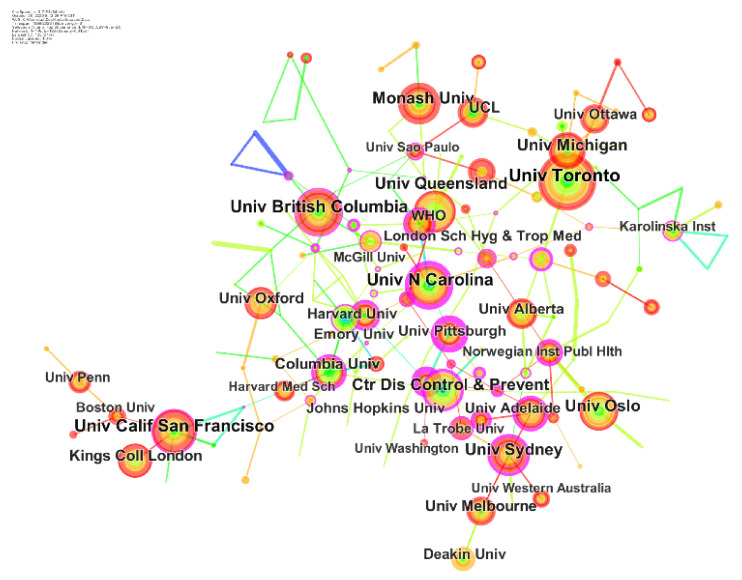
Collaborative networks of institutes.

Figure 5 is a collaborative map of institutions in maternal health research in the environment of new media. In the figure, the node represents the issuing institution, and the name of each institution is indicated in black text in the figure. The larger the font, the more the amount of the issuing institution. Totally, the 2270 articles were published by 148 research institutions. The leading institutions were in America, including the University of Toronto, the University of California and the University of North Carolina. Then, university systems were also located in Australia, including the University of Monash, the University of Queens-land, and the University of Technology Sydney. Meanwhile, the line between nodes represents the cooperative relationship between institutions. The thicker the line, the more frequent the cooperation between institutions. In this study, all the institutions engaged in maternal health research under the new media environment had cooperative relationships. The density of the entire network was only 0.0155, indicating that the cooperation between institutions needed to be strengthened. Notably, the quality of publications could not be ignored in scientometrics analysis. Among all institutions, Eunice Kennedy Shriver Natl Inst Child Hlth & Hum, the University of North Carolina at chapel hill, WHO, and Nottingham Stan Stellenbosch university had higher centrality and played crucial roles in promoting research cooperation. The top 10 countries and institutions are shown in Table 1.

### 3.3. Cited Reference Analysis

**Figure 6:**
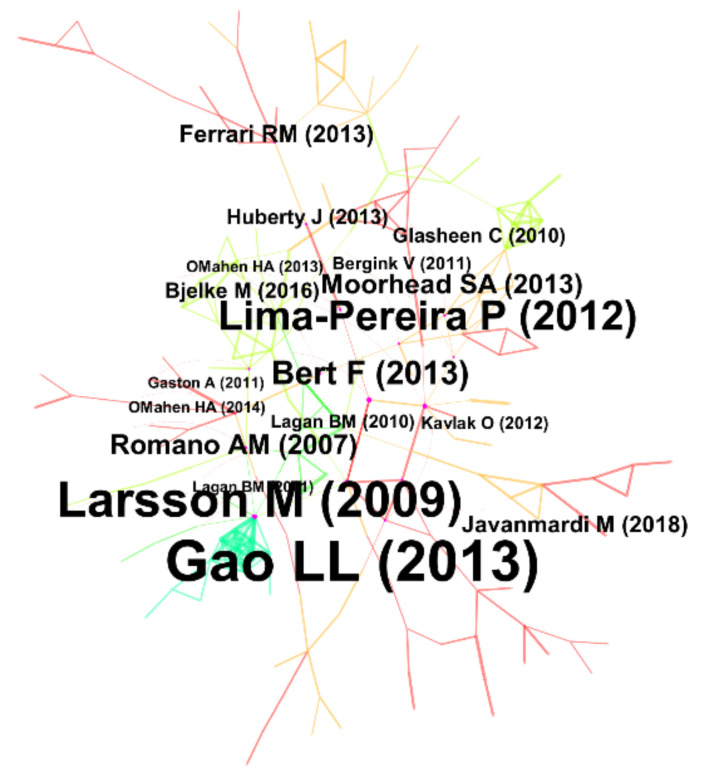
The network of highly cited references.

In the bibliometric analysis, cited references form the knowledge base [29]. Figure 6 is the map of highly cited references on maternal health in the new media environment. In the figure, nodes represent the first author and publication time of highly cited references. The larger the font, the higher the citation frequency of this article. At the same time, the line between nodes represents the relationship between various publications. The thicker the line, the stronger the relationship. Furthermore, this study selected 15 publications with centrality greater than 0.1 as the dataset for full-text analysis in Table A1 of the Appendix A. Among them, articles published by Gao Ling-ling [30] and Larsson Margareta [31], in 2013 and 2009, had 0.37 and 0.33 centrality respectively. Strikingly, Larsson Margareta’s research had a burst intensity as high as 10.96 in Table A2 of the Appendix A, which was an essential reference for the study of maternal health in the context of new media, especially in information acquisition behavior [32]. In addition, the two research papers of Lagan Briege M had high burst intensity and long duration between 2013 and 2021, which had been widely concerned by researchers.

As the number of publications continues to increase, a new paper might change the structure of the existing research network [32]. Structural variation analysis may measure the potential impact on the existing network structure and reveal the mechanism of scientific development, which was also the lack of traditional bibliometrics studies [29]. In the analysis of structural variation, the modularity change rate, the change of inter-cluster connection, and the change of centrality distribution all could be used to measure the academic influence. Strikingly, ΔM is used in the analysis of CiteSpace by Professor Chen Chaomei, who developed the software and explained the calculation method in detail [33]. In this paper, ΔM was also chosen why the higher the value of ΔM was, the greater the potential influence of the new publication on the current network was. 

In general, among the 2270 articles, 32.36% of the articles had a modularity change rate greater than zero. The top 10 articles with the most influence are shown in Table A3 of the Appendix A. Firstly, the highest article was published in 2008. The paper focused on maternal depression, fertility desire, anorexia, and other psychosocial factors.The authors studied their influence on maternal health and neonatal diseases, which varied with different medical insurance types and races. Another example was the effect of maternal diet on the fitness of infants [34]. At the same time, this article published in the last five years focused on neonatal nutrition. That article confirmed that providing health guidance and counseling services to new parents was conducive to forming good eating habits in infants early, effectively preventing obesity and overweight in newborns. 

### 3.4. Research Frontiers

As the carrier of science, vocabulary can reveal the development and change of science [35]. This study also displayed the topics based on the keywords in each article (Table 2). At the same time, citation bursts were available, and it could better reflected the research frontiers [36]. This research drew a burst map of the top 20 terms (Table 3). In the diagram, the blue line represents the entire research period, and the red displays the duration of the burst, with the beginning and end years of the activity as both ends of the endpoint. 

As can be seen from Table 3, the burst is generally mainly distributed from 2000 to 2020. Firstly, from 2000 to 2012, “infant” had been the focus of researchers, and the burst intensity of 15.65 was the highest. Meanwhile, topics related to macrosomia, such as “weight”, “obesity”, and “body mass index”, also received more attention from 2006 to 2014. Secondly, between 2014 and 2017, researchers focused more on “prevention”, which offered new ideas for promoting maternal health. In 2015, "Internet" also became a hotspot. Finally, between 2017 and 2020, research started to move out of the comfort zone. Not only had the trend of going back to the source, but the risk factors affecting maternal health had been paid attention to. Meanwhile, health education for pregnant women and psychological problems were also slowly breaking the previous research’s bottleneck.

### 3.5. Research Hotspots

**Figure 7:**
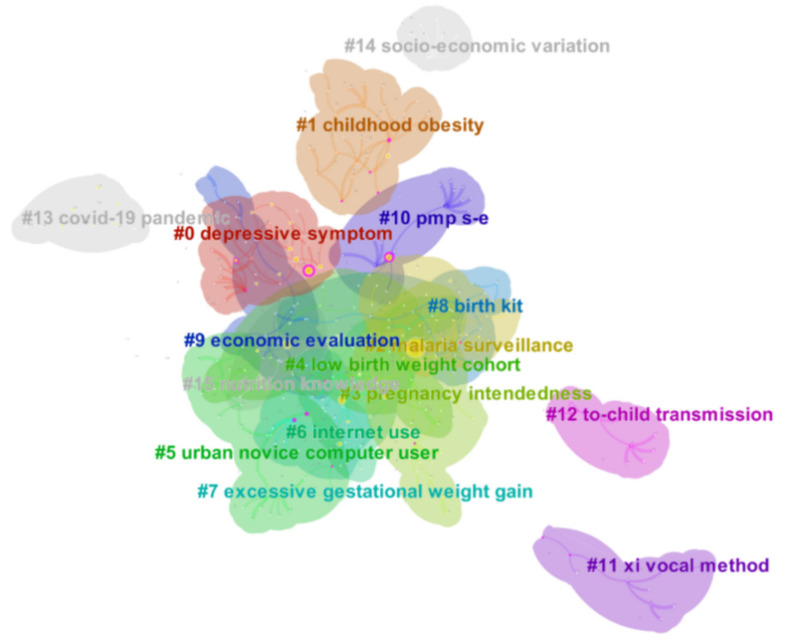
The hotspots cluster.

Based on the clustering results of structural variation analysis of cited references, this part aims to analyze the current research hotspots. In the cited references clusters, the present study mainly distributed in three areas: psychological, nutrition, and technology. The research hotspots mainly focused on maternal depressive and fertility intentions, maternal and child nutrition, production equipment, reproductive technology, the impact of malaria surveillance, and the outbreak of the COVID-19. (Figure 7, Supplementary Material Figure S1)

Firstly, studies on maternal mental health in the new media environment included clusters #0 and #3, which lasted from 2001 to 2019. Maternal depression might be appropriately considered a health problem [37]. This hotspot focused on postnatal depression, mental health, pregnancy intendedness, maternal psychosocial factors, women’s expectations, influencing factors, and pain relief. The article published in 2008 is the most representative, as shown in Supplementary Material Figure S2. The solid pink line represents the research structure before the paper’s publication, while the dotted red line indicates the influence of the paper on the network structure. This paper linked psychosocial factors such as pregnant women’s fertility with low birth weight [38]. It was an essential reference for the mental health of pregnant women in the new media environment.

Secondly, studies on maternal and neonatal nutrition in the new media environment include Clusters #1, #4, and #7, but the popularity declined in the past three years. It focused on childhood obesity, food neophobia, maternal feeding style, children’s body size, low birth weight cohort, economic factors, gestational weight gain, and postpartum weight loss. This article published in 2011 is typical, as shown in Supplementary Material Figure S3. This article takes a cesarean section of pregnant women as the research topic and emphasizes the use and monitoring of computers based on the traditional network structure [39].

Thirdly, research on reproductive technology in the new media environment included clusters #2, #5, #6, #8, and #15, and this hotspot lasted the longest. Main keywords included malaria surveillance, technology-based peer support, urban novice computer user, web-based survey, Internet use, and safe childbirth. In developed countries, most maternal deaths were mainly due to anesthesia and cesarean section complications, so improved production equipment and technology were also essential to ensure maternal security [40]. This article (Supplementary Material Figure S4) is published in 2015, with a 13.946 impact factor. And it highlighted the clinical manifestation types of neonatal preterm birth syndrome. Thus, new connections were also being established in clusters #2, #3, #4, and #8, which significantly impacted traditional network architecture [41].

Simultaneously, in the COVID-19, researchers also began to pay attention to the influence on maternal health, mainly including the COVID-19, related factors, cross-sectional study, mental health, developmental outcome, longitudinal cohort study, and COVID-19 lockdown. COVID-19 could lead to severe morbidity and perinatal death in pregnant women, so there is a need for enhancing surveillance to avoid maternal and even neonatal infection [42,43].

## 4. Discussion

### 4.1. Principal Results

This study aimed to investigate the influence of the new media on maternal fitness and the hotspots arising in academics over a period of nearly 20 years, then vividly present them with scientometrics approach. The findings indicated that the amount of publications and citations has grown substantially since 2008, and the increase was particularly notable by 2020. The reason why is after the global economic crisis, governments around the world not only focused on economic recovery, but also payed more attention to the quality of development. Unfortunately, due to nearly five years of growth, we did not achieve the ideal value of exponential growth model, the growth of publications did not follow the Law of Price. It suggests that the governments need to emphasize the importance of maternal health in policies, encourage maternal health research in science and technology under the new media environment, arousing the enthusiasm of researchers and corporations. However, there was a clear difference in the distribution, the dominant power of studies was in the United States, and the University of Toronto also occupied an important position. The reason might be that maternal mortality is descending globally, but the United States is ascending continuously [39]. Particularly from 2008 to 2009, maternal mortality under the age of 40 per 100,000 population had increased 25% [44], which might have attracted careful consideration of governments and academics. Furthermore, the 2008 US election campaign has demonstrated the important role new media played in information transmission [45]. It has been speculated that, internationally, publications on new media has also set the 2008 demarcation point, after which it has begun to grow significantly [46]. However, the cooperation between China and the United States, the United Kingdom also needs to be strengthened in the future. Another finding indicated that mental health emerged in 2001 and gradually leveled off in 2019. This may be explained why the new media environment is a double-edged sword. Although a large amount of redundant information might be misleading for the pregnant woman, social software such as Facebook or WeChat has made communication more convenient and beneficial for alleviating antenatal or postpartum maternal anxiety. To illustrate, the Chinese two-child policy did not produce the desired effect [47,48] why the government also needs to relieve maternal anxiety and depression to improve childbearing intentions [49].

Co-cited reference shows that Larsson M, Lagan BM, and Tiedje L attracted the attention of many researchers in recent years. The centrality analysis and burst analysis were relatively single, mainly focusing on pregnant women’s health information searching behavior in the new media environment. Instead, the research contents of structural variation analysis is diverse relatively. For example, publications on mental health and newborn nutrition have high potential impact. This also indicates that only a certain method of scientometrics analysis may lead to a slight bias in the results. However, we ingeniously combined co-cited analysis, burst analysis and structural variation analysis to explore the research hotspots and frontiers of maternal health in the new media environment, which is a innovative point to a certain extent. Interestingly, articles or authors that learned the critical node articles and innovate had a high modularity rate, positively influencing the research network. So, it is critical to establish new connections based on the existing research network structure.

### 4.2. Past Studies and Future Directions

Maternal health, as an important strategy to improve social health, has attracted much attention from the international community. Compatible with the ever-increasing number of publications, which have analyzed the current research status on maternal health with scientific methods. The development of new media has facilitated people’s lives. However, the research regarding maternal health in the new media environment based on an international perspective was little. Similar to the results of this study, a lancet commission reported that challenges in improving maternal and neonatal health in China focused on delivery safety, maternal and neonatal nutrition, mental health, and stillbirth [50]. Studies in the United States showed that maternal research gradually shifted from physical health and weight to mental health and satisfaction, conforming to the predictions that depression was one of the critical causes of the global burden of diseases [47,48]. This research also found neonatal nutrition and the resulting unhealthy conditions (e.g., low birth weight or macrosomia) may be one of the hotspots. A recent trial study in Timor Leste also showed that maternal food intake affected children’s nutrition and weight [51]. Furthermore, preventing excessive weight gain in mothers and adjusting post-weaning diets could help reduce childhood obesity worldwide [52].

This study used structural variation analysis to explore the current research hotspots. And in the analysis of high-cited reference, the modularity rate was used to measure the potential impact of articles, which is different from previous scientometrics analysis. On the other hand, there were several limitations, such as the quality of data and the stability of software runs, it was also the future research direction. It is also possible that databases such as PubMed and Scopus may have higher quality publications, but due to the limitation of study manpower, this study only selected Web of Science to collect publications and used CiteSpace to remove duplicates, which might have biased our analysis results. To address this problem, future research would like to explore appropriate seed articles and use the cascade extension function to screen out high-quality publication’s collection [29]. Besides, this study will also verify if the parameters change, the study will get the same conclusion, or if there is a final threshold that makes the result stable. Despite these limitations, this study provides several key insights to understand maternal fitness in the new era and master important approaches in future research.

## 5. Conclusions

This scientometrics study shows a rapid increase since 2008 in the number of publiactions on maternal fitness in the new media environment. However, in terms of countries, institutions as well as high-impact articles, the research power is mainly distributed in North America. Specifically, the United States played a leading role with a comparatively large amount of publications, while the University of Toronto was better than other institutions. Maternal depression, maternal and infant nutrition, low birth weight, macrosomia, and delivery technique and facilities had received extensive attention from the scientific research personnel. Future research perspectives may also shift from physiological disorders to mental health and explore the potential of advanced technologies in improving maternal health. Besides, this study also offers sigshts on the research method and provides valuable information for researchers to find their research directions. 

## Figures and Tables

**Table 1:** Top countries, institutes according to the number of publications.

Ranking	Frequency	Country	Frequency	Institution
1	905	The United States	52	University of Toronto
2	305	Australia	42	University of California
3	277	The United Kingdom	41	University of North Carolina
4	216	Canada	38	University of British Columbia
5	121	Netherlands	37	University of Monash
6	84	China	34	University of Queensland
7	76	Brazil	34	University of Oslo
8	68	Switzerland	34	University of Michigan
9	67	Spain	34	US Center for Disease Control and Control
10	65	Sweden	30	University of Technology Sydney

**Table 2:** Top 10 high-frequency keywords and high centrality keywords.

Ranking	High-Frequency Keywords			High Centrality Keywords		
	Keyword	Frequency	Year	Keyword	Centrality	Year
1	pregnancy	569	1998	social support	0.40	2003
2	health	316	2001	intervention	0.38	2006
3	risk	235	2000	childhood obesity	0.31	2006
4	internet	165	2002	postpartum overweight	0.26	2006
5	care	158	2007	women	0.24	1998
6	intervention	155	2008	cancer	0.22	2002
7	mother	152	2004	nutrition	0.21	2007
8	children	145	1999	HIV infection	0.20	2006
9	prevalence	143	2004	information	0.20	2005
10	pregnant women	141	2004	community	0.19	2005

**Table 3:** Top 20 Keywords with the Strongest Citation Bursts.

Ranking	Keywords	Year	Strength	Begin	End	1998–2021
1	infant	1998	15.65	2000	2012	▂▂ ▃▃▃▃▃▃▃▃▃▃▃▃▃ ▂▂▂▂▂▂▂▂▂
2	overweight	1998	8.57	2006	2014	▂▂▂▂▂▂▂▂▂ ▃▃▃▃▃▃▃▃ ▂▂▂▂▂▂▂
3	children	1998	18.22	2008	2017	▂▂▂▂▂▂▂▂▂▂ ▃▃▃▃▃▃▃▃▃▃ ▂▂▂▂
4	mortality	1998	7.31	2008	2014	▂▂▂▂▂▂▂▂▂▂ ▃▃▃▃▃▃▃ ▂▂▂▂▂▂▂
5	exposure	1998	7.18	2009	2012	▂▂▂▂▂▂▂▂▂▂▂ ▃▃▃▃ ▂▂▂▂▂▂▂▂▂
6	body mass index	1998	6.00	2009	2011	▂▂▂▂▂▂▂▂▂▂▂ ▃▃▃ ▂▂▂▂▂▂▂▂▂▂
7	adolescent	1998	5.52	2009	2012	▂▂▂▂▂▂▂▂▂▂▂ ▃▃▃▃ ▂▂▂▂▂▂▂▂▂
8	low birth weight	1998	9.11	2010	2013	▂▂▂▂▂▂▂▂▂▂▂▂ ▃▃▃▃ ▂▂▂▂▂▂▂▂
9	perception	1998	8.50	2010	2014	▂▂▂▂▂▂▂▂▂▂▂▂ ▃▃▃▃▃ ▂▂▂▂▂▂▂
10	preeclampsia	1998	7.98	2011	2012	▂▂▂▂▂▂▂▂▂▂▂▂▂ ▃▃ ▂▂▂▂▂▂▂▂▂
11	randomized controlled trial	1998	7.03	2011	2014	▂▂▂▂▂▂▂▂▂▂▂▂▂ ▃▃▃▃ ▂▂▂▂▂▂▂
12	stress	1998	5.22	2012	2013	▂▂▂▂▂▂▂▂▂▂▂▂▂▂ ▃▃ ▂▂▂▂▂▂▂▂
13	behavior	1998	10.63	2013	2017	▂▂▂▂▂▂▂▂▂▂▂▂▂▂▂ ▃▃▃▃▃ ▂▂▂▂
14	childbirth	1998	9.13	2013	2017	▂▂▂▂▂▂▂▂▂▂▂▂▂▂▂ ▃▃▃▃▃ ▂▂▂▂
15	prevention	1998	15.78	2014	2017	▂▂▂▂▂▂▂▂▂▂▂▂▂▂▂▂ ▃▃▃▃ ▂▂▂▂
16	united states	1998	8.25	2014	2015	▂▂▂▂▂▂▂▂▂▂▂▂▂▂▂▂ ▃▃ ▂▂▂▂▂▂
17	internet	1998	10.18	2015	2016	▂▂▂▂▂▂▂▂▂▂▂▂▂▂▂▂▂ ▃▃ ▂▂▂▂▂
18	attitude	1998	12.28	2017	2019	▂▂▂▂▂▂▂▂▂▂▂▂▂▂▂▂▂▂▂ ▃▃▃ ▂▂
19	knowledge	1998	11.10	2018	2021	▂▂▂▂▂▂▂▂▂▂▂▂▂▂▂▂▂▂▂▂ ▃▃▃▃
20	mental health	1998	17.80	2019	2021	▂▂▂▂▂▂▂▂▂▂▂▂▂▂▂▂▂▂▂▂▂ ▃▃▃

The blue bar is the research phase, and the red bar is the time period of keyword burst.

## Data Availability

For data availability, please contact Yinghua Xie.

## Supplementary Materials

The following are available online at https://www.mdpi.com/article/10.3390/ijerph182413095/s1, Figure S1: The time line of hotspot clustering, Figure S2: Structural variation arising from representative literature on maternal mental health, Figure S3: Structural variation arising from representative literature on maternal and neonatal nutrition, Figure S4: Structural variations arising from representative literature on reproductive technology and devices.

## Appendix A

**Table A1:** Highly cited references (Sort by centrality).

Ranking	Centrality	Author	Year	DOI
1	0.37	Gao Ling-ling	2013	10.1016/j.midw.2012.07.003
2	0.33	Larsson Margareta	2009	10.1016/j.midw.2007.01.010
3	0.27	Lima-Pereira Patricia	2012	10.1111/j.1365-2702.2011.03910.x
4	0.22	Bert Fabrizio	2013	10.1136/jech-2013-202584
5	0.18	Moorhead S. Anne	2013	10.2196/jmir.1933
6	0.18	Romano Amy M	2007	10.1624/105812407X244903
7	0.16	Ferrari Renee M	2013	10.1016/j.pec.2013.01.011
8	0.15	Javanmardi Marzieh	2018	10.4103/ijnmr.IJNMR_82_17
9	0.14	Huberty Jennifer	2013	10.1007/s10995-012-1160-2
10	0.14	Bjelke Maria	2016	10.1016/j.midw.2016.06.020
11	0.13	Glasheen Cristie	2010	10.1007/s00737-009-0109-y
12	0.12	Lagan Briege M	2010	10.1111/j.1523536X.2010.00390
13	0.12	Bergink Veerle	2011	10.1016/j.jpsychores.2010.07.008
14	0.11	O’Mahen H. A.	2014	10.1017/S0033291713002092
15	0.11	Kavlak Oya	2012	10.3109/17538157.2012.710686

**Table A2:** Top 25 References with the Strongest Citation Bursts.

Ranking	DOI	Year	Strength	Begin	End	1998–2021
1	10.1016/j.midw.2007.01.010	2009	10.96	2009	2018	▂▂▂▂▂▂▂▂▂▂▂ ▃▃▃▃▃▃▃▃▃▃ ▂▂▂
2	10.1016/S0140-6736(10)60518-1	2010	3.98	2010	2015	▂▂▂▂▂▂▂▂▂▂▂▂ ▃▃▃▃▃▃ ▂▂▂▂▂▂
3	10.1038/ijo.2008.240	2008	3.86	2010	2012	▂▂▂▂▂▂▂▂▂▂ ▂▂ ▃▃▃ ▂▂▂▂▂▂▂▂▂
4	10.1624/105812407X191506	2007	3.83	2010	2015	▂▂▂▂▂▂▂▂▂ ▂▂▂ ▃▃▃▃▃▃ ▂▂▂▂▂▂
5	10.1289/ehp.9478	2006	3.67	2010	2015	▂▂▂▂▂▂▂▂ ▂▂▂▂ ▃▃▃▃▃▃ ▂▂▂▂▂▂
6	000308131701788	2010	10.00	2013	2018	▂▂▂▂▂▂▂▂▂▂▂▂ ▂▂▂ ▃▃▃▃▃▃ ▂▂▂
7	10.1016/j.drugalcdep.2010.01.007	2010	7.86	2013	2018	▂▂▂▂▂▂▂▂▂▂▂▂ ▂▂▂ ▃▃▃▃▃▃ ▂▂▂
8	10.1371/journal.pmed.1000097	2009	6.39	2013	2018	▂▂▂▂▂▂▂▂▂▂▂ ▂▂▂▂ ▃▃▃▃▃▃ ▂▂▂
9	10.1097/ACM.0b013e3181a8144d	2009	6.21	2013	2018	▂▂▂▂▂▂▂▂▂▂▂ ▂▂▂▂ ▃▃▃▃▃▃ ▂▂▂
10	10.1111/j.1523-536X.2011.00488.x	2011	4.79	2013	2021	▂▂▂▂▂▂▂▂▂▂▂▂▂ ▂▂ ▃▃▃▃▃▃▃▃▃
11	10.1007/s00228-009-0744-2	2010	4.45	2013	2018	▂▂▂▂▂▂▂▂▂▂▂▂ ▂▂▂ ▃▃▃▃▃▃ ▂▂▂
12	10.1007/s10654-005-6030-4	2006	4.16	2013	2015	▂▂▂▂▂▂▂▂ ▂▂▂▂▂▂▂ ▃▃▃ ▂▂▂▂▂▂
13	10.1016/S0140-6736(12)60107-X	2012	4.13	2013	2018	▂▂▂▂▂▂▂▂▂▂▂▂▂▂ ▂ ▃▃▃▃▃▃ ▂▂▂
14	10.1186/1471-2296-10-34	2009	3.54	2013	2018	▂▂▂▂▂▂▂▂▂▂▂ ▂▂▂▂ ▃▃▃▃▃▃ ▂▂▂
15	10.1007/s10995-012-1160-2	2013	5.84	2016	2021	▂▂▂▂▂▂▂▂▂▂▂▂▂▂▂ ▂▂▂ ▃▃▃▃▃▃

The blue bar is the research phase, and the red bar is the time period of keyword burst.

**Table A3:** Top 15 publications with potential transformational significance.

Ranking	∆M	Year	DOI
1	98.30	2008	10.1016/j.socscimed.2007.11.039
2	95.54	2011	10.1057/sth.2010.12
3	95.04	2012	10.1186/1471-2393-12-25
4	94.26	2015	10.1001/jamapediatrics.2014.3040
5	94.26	2013	10.1080/10810730.2013.768731
6	93.93	2011	10.1038/ejcn.2011.16
7	93.62	2007	10.1111/j.1740-8709.2007.00108.x
8	93.19	2009	10.1016/j.jmwh.2008.09.006
9	92.63	2017	10.1186/s12889-017-4731-8
10	92.56	2011	10.1177/1049732311404903
11	92.56	2010	10.1038/ijo.2009.275
12	92.19	2015	10.1186/s12884-015-0784-9
13	90.89	2010	10.1038/ijo.2010.10
14	90.70	2012	10.1097/QAD.0b013e328359590f
15	90.70	2012	10.1093/alcalc/agr145

∆M: Modularity change rate.
