# Supplementary material for: Mapping Maternal Health in the New Media Environment: A Scientometric Analysis
Source: Int J Environ Res Public Health. 2021 Dec 11;18(24):13095. doi: 10.3390/ijerph182413095 (PMC8700903; doi:10.3390/ijerph182413095)
Supplement: Supplementary file 1 [file ijerph-18-13095-s001.zip › ijerph-1455087-supplementary.pdf]

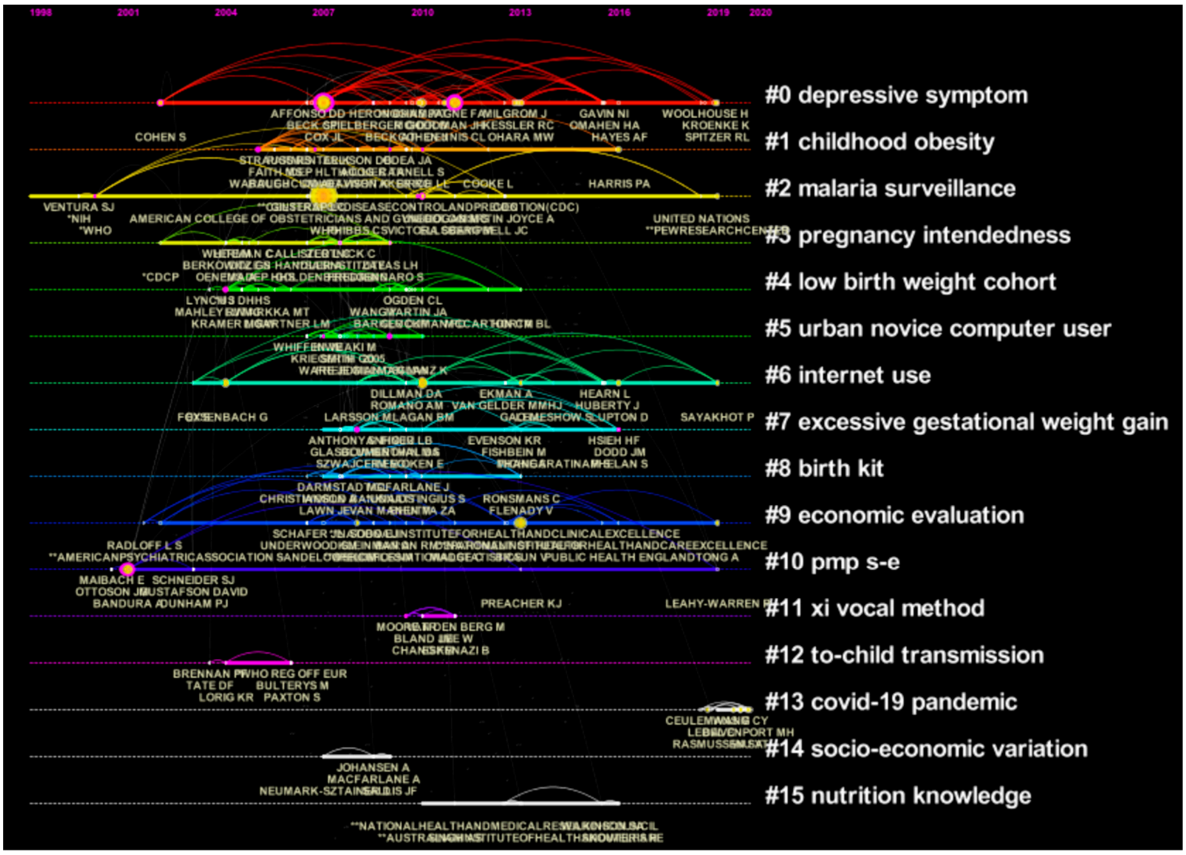

Figure S1: The time line of hotspot clustering.

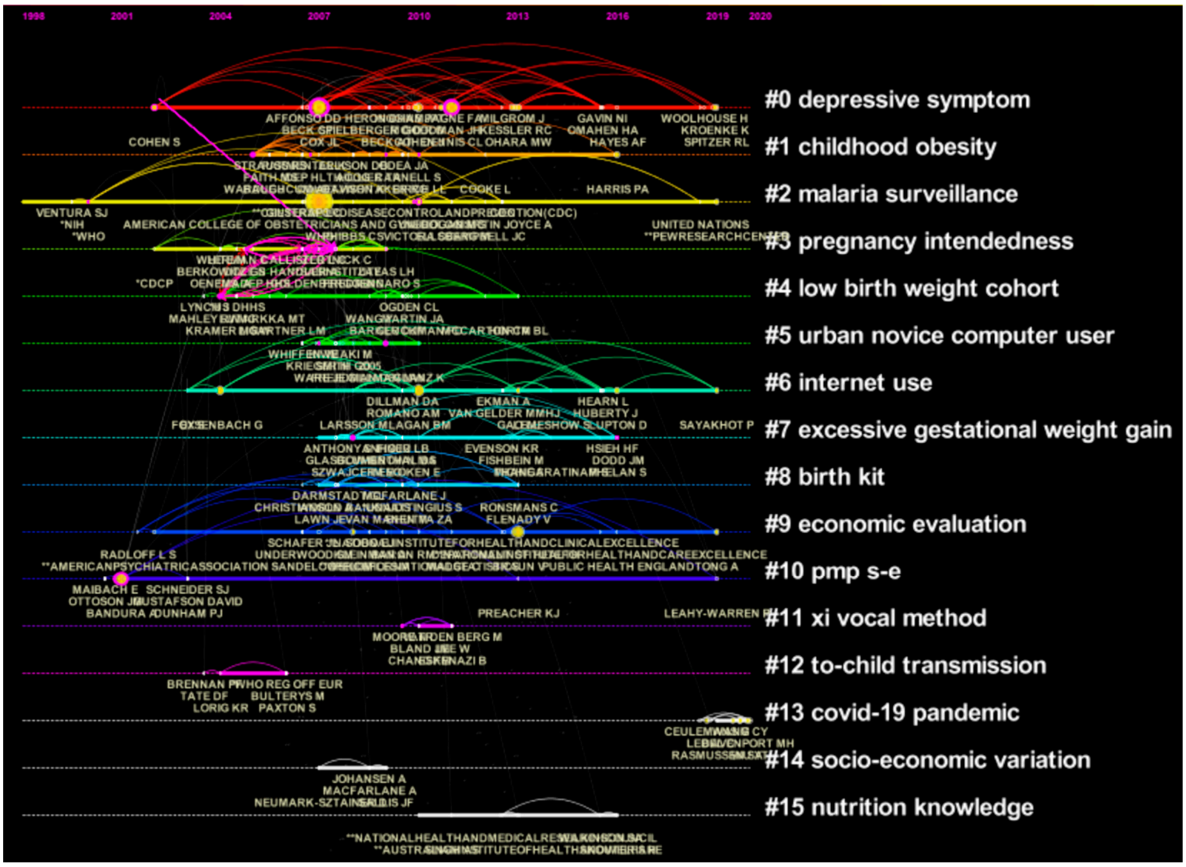

Figure S2: Structural variation arising from rep-resentative literature on maternal mental health.

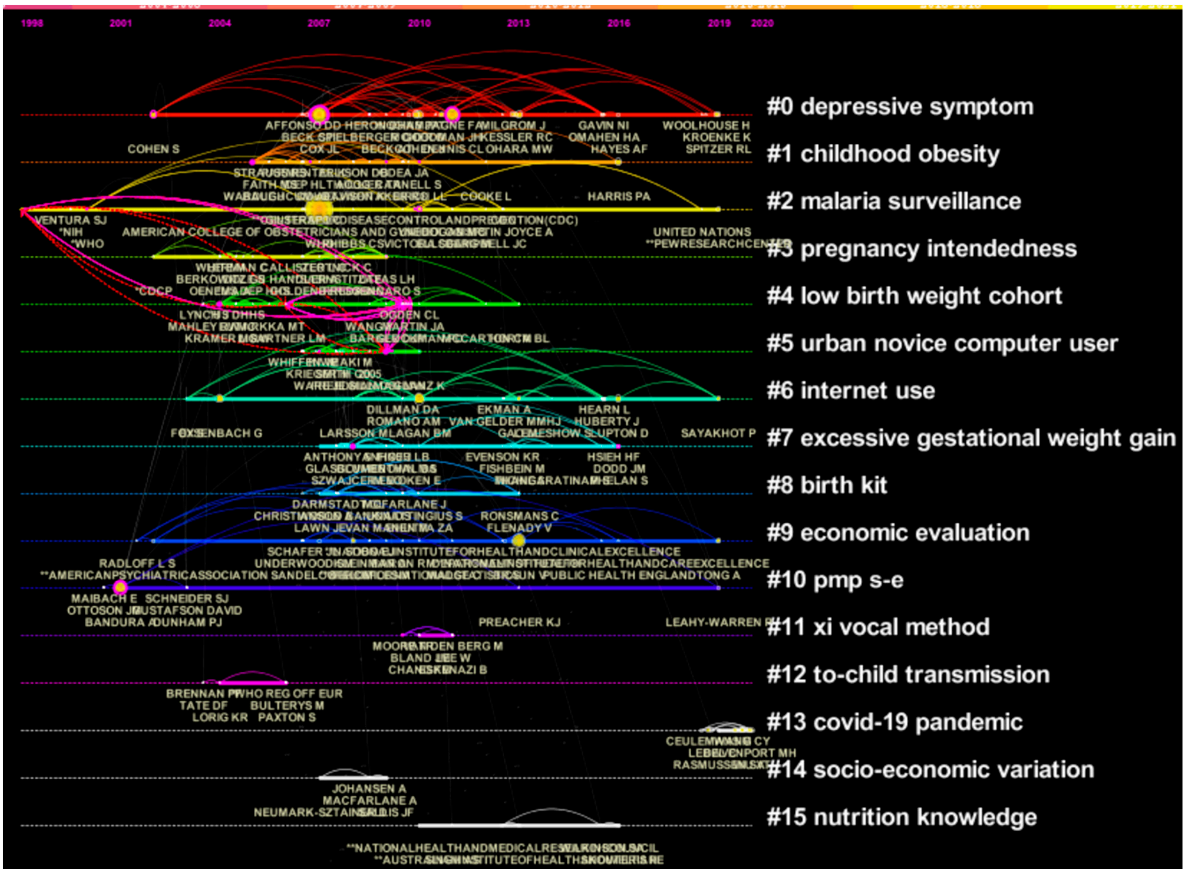

Figure S3: Structural variation arising from representative literature on maternal and neonatal nutrition.

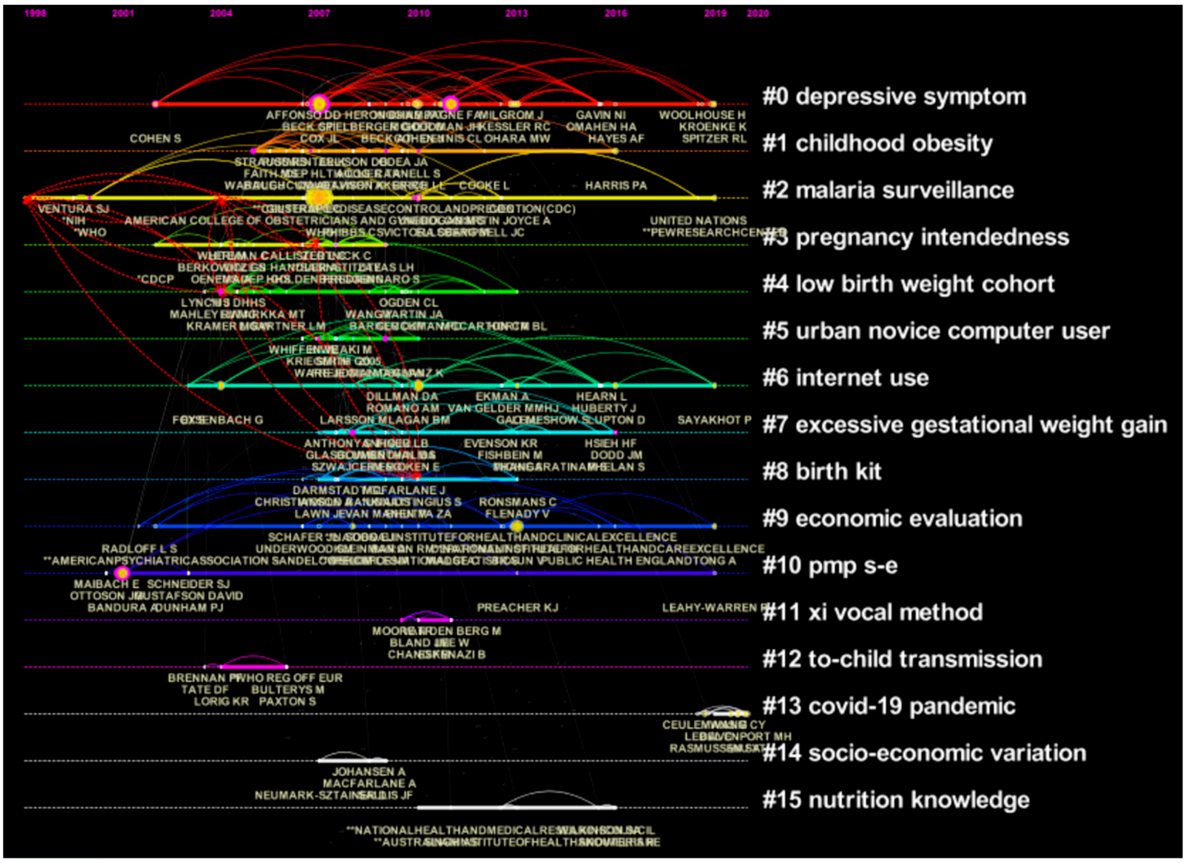

Figure S4: Structural variations arising from representative literature on reproductive technology and devices.
